# Memory precision and age differentially predict the use of decision-making strategies across the lifespan

**DOI:** 10.1038/s41598-023-44107-5

**Published:** 2023-10-09

**Authors:** Sharon M. Noh, Umesh K. Singla, Ilana J. Bennett, Aaron M. Bornstein

**Affiliations:** 1 Department of Cognitive Sciences, The University of California, Irvine, Irvine, CA USA; 2 Princeton Neuroscience Institute, Princeton, NJ USA; 3 Department of Computer Science and Engineering, University of California, San Diego, La Jolla, CA USA; 4 Department of Psychology, The University of California, Riverside, Riverside, CA USA; 5 Center for the Neurobiology of Learning and Memory, The University of California, Irvine, Irvine, CA USA

**Keywords:** Human behaviour, Psychology

## Abstract

Memory function declines in normal aging, in a relatively continuous fashion following middle-age. The effect of aging on decision-making is less well-understood, with seemingly conflicting results on both the nature and direction of these age effects. One route for clarifying these mixed findings is to understand how age-related differences in memory affect decisions. Recent work has proposed *memory sampling* as a specific computational role for memory in decision-making, alongside well-studied mechanisms of reinforcement learning (RL). Here, we tested the hypothesis that age-related declines in episodic memory alter memory sampling. Participants (total N = 361; ages 18–77) performed one of two variants of a standard reward-guided decision experiment with additional trial-unique mnemonic content and a separately-administered task for assessing memory precision. When we fit participants’ choices with a hybrid computational model implementing both memory-based and RL-driven valuation side-by-side, we found that memory precision tracked the contribution of memory sampling to choice. At the same time, age corresponded to decreasing influence of RL and increasing perseveration. A second experiment confirmed these results and further revealed that memory precision tracked the specificity of memories selected for sampling. Together, these findings suggest that differences in decision-making across the lifespan may be related to memory function, and that interventions which aim to improve the former may benefit from targeting the latter.

## Introduction

It is widely accepted that memory, at least that for individual experiences, declines with age^1,2^. The results on decision-making, however, are more mixed. While some studies identify a decrease in the ability of older individuals (and/or those experiencing cognitive decline) to engage in multi-step planning^3–5^—a function which, like episodic memory, is critically linked to an intact hippocampal formation^6^—others report that older individuals have spared, and sometimes improved, decision-making abilities, such as in a resistance to sunk costs^7^. Still others have identified increases in choice randomness with age^8^, which at first blush seems incompatible with reports of increasing perseveration—repetition of the same choice irrespective of learned values^9–11^. The field has yet to settle on a unifying explanation for these seemingly disparate phenomena.

One route to a synthesis may be via understanding the role of memory in decisions. A number of findings have shown that memories for individual experiences can bias decision-making even in repeated selection tasks, where individuals gain extensive experience with the probabilistic outcomes of choice options^12,13^. The role of memory in decisions via *sampling* (i.e. the selective retrieval of memories of similar past choices during decision-making, to estimate the value of one action or another^14,15^) has been shown to capture the influence of recent rewarding experiences on choice^16^, while also serving an adaptive role in decisions when familiarity with an environment is low or uncertainty about the structure of the environment is high^17^. To illustrate the difference between these routes to value estimation, consider choosing between two restaurants: One, an old favorite that you have been to many times; the other, a brand-new shop of a well-known type—e.g. a slice pizza stand. For the former, you can rely on your repeated experiences to estimate the value of dining there tonight. For the latter, you must draw on memories of similar experiences, and extrapolate from there. When evaluating repeated choice options, and assuming that recent memories are more likely to be retrieved, these two approaches give similar results on average, but can diverge, especially when the content of memory contains more than just recent experience.

Building on the findings that each system contributes to behavior, work has begun to examine what factors influence the relative use of one or the other. A recent study in young adults observed that the relative uncertainty of values estimated using memory sampling and reinforcement learning indexed how strongly choices depend on each system on a given trial^18^. In other words, when one system had more variable estimates of option values on a given trial, it had less influence on choice. This finding concords with theoretical frameworks in which multiple learning systems contribute to behavior in proportion to the relative precision of their estimates of upcoming stimuli^19–22^. An open question is whether this mixture of decision processes is altered across the lifespan, perhaps as a result of relative declines in the fidelity of memory representations or neural circuits communicating between valuation systems and action selection regions^23–26^.

Therefore, the precision of memory representations may play a critical role in its influence on choice. A link between memory precision and memory-guided decision-making may be crucial to understanding age-related differences in choice patterns. This is because a key aspect of memory that declines with aging is the ability to *pattern separate*, or represent similar mnemonic stimuli with sufficiently distinct, non-overlapping memory traces^5,27–30^. Individuals with impaired pattern separation may encode experiences in a manner that leads to impaired retrieval, perhaps due to the relative imprecision of these representations. Pattern separation is used to store unique memories of even highly similar information without interference, and age-related reductions in pattern separation result in less precise, lower-fidelity memory in older adults.

Here, we examined whether a behavioral measure of pattern separation—mnemonic discrimination ability in the Mnemonic Similarity Task (MST;^27^)—indexed the contribution of memory sampling to behavior in a repeated choice task where outcomes were linked with trial-unique memoranda^16,31^. A lifespan sample of individuals (ages 18–77; total *N* = 361) performed a series of choices between three options, each of which had steadily varying probabilities of paying reward. After selecting an option, they were then asked to encode a trial-unique object photograph in concert with one of six scene images. Their ultimate reward in the task depended both on their ability to identify and track the winningest choice option, and also on their ability to later successfully recall the object and scene pair that was associated with a randomly-selected subset of the choice outcomes. Critically, the experiment included a second phase during which individuals were presented with memory probes that incidentally reminded them of past choices. These probes have previously been shown to influence subsequent choice via intrusions of value representations linked to the reminded trials^16,31^ and associated contexts^31^ in younger adults. It is important to note that this effect of incidental reminder probes on choice is not adaptive—individuals would do best to attend only to recent rewards, and not the values reminded by the probes. Therefore, this aspect of the task serves as a strong test of the hypothesis that action selection incorporates value information from recent memory, independent of or alongside continual reinforcement learning-based approaches^17,21,32^. To date, memory sampling has not been studied in older adults.

We fit each participant’s series of choices with a computational model implementing both memory sampling and reinforcement learning, and measured the degree to which the values estimated by each process influenced the individual’s choice behavior. We then examined the relationship between their reliance on each process and their ability to discriminate similar objects during the MST, administered in a separate session. We predicted that, independent of age, the latter measure of mnemonic discrimination ability would index the average precision of an individual’s trial-level outcome memories in the choice task, and thus their use of memory sampling. We further predicted that age would negatively influence choice performance.

Consistent with theories of uncertainty-weighted arbitration between decision systems^19–21^, which predict that decision systems with more precise estimates will have greater influence on choices, we found that higher memory precision predicted a greater influence of memory sampling-derived values on behavior, while not affecting an individual’s sensitivity to values learned by recent reinforcement. At the same time, age increased the appearance of “noisy” choices, reflected in a decreased reliance on reinforcement learning, while also increasing the influence of perseveration.

To further understand how memory precision altered the influence of memory content on decisions, we next performed a variant of the experiment in which individuals were presented with perceptually aliased contexts—three matched pairs of scenes with similar visual content, but distinct choice-reward associations. We then tested whether individual memory precision corresponded to the influence on choice of the *target* context (the scene image associated with the memory probe) or the *gist* context (the target as well as its matched pair lure context, which had distinct choice-reward values). Consistent with the idea that uncertainty in memory representations is itself sample-based, rather than inherent to the representation, we found that lower memory precision was associated with a greater influence of gist-level memories.

Taken together, these findings support the idea that age-related declines in memory precision lead to changes in decision profiles, which can be decomposed with normatively-motivated process models. They further suggest that individuals who exhibit noisier choices with age may benefit from interventions that target the precision of their memory representations. Lastly, our results shed light on the fundamental mechanisms that guide the weighting of different decision strategies in individuals across the lifespan.

## Methods

Experiments 1 and 2 were identical in procedure other than the background scene images (*contexts*) presented during the learning phase of the three-armed bandit task. As such, we have combined the method across both experiments and specified the differences across experiments where applicable.

### Participants

Participants across the lifespan were recruited online on Amazon Mechanical Turk via the CloudResearch interface (*N* = 295, ages 19–77, mean(sd) age = 46.7(16.5), 144 Male, 149 Female, 1 Other, 1 Unknown) and in-person (*N* = 156, ages 18–45, mean(sd) age = 24.2(7.0), 56 Male, 97 Female, 1 Other). The majority of data collection occurred early in the COVID-19 pandemic, which limited our ability to collect in-person data, particularly those in older age groups. Though recent work has found that online older adult participants evince highly similar memory profiles to those collected in person^33^, we instituted a multi-tiered quality control system to ensure commensurate data. Specifically, for online samples, we set eligibility filters through Amazon Mechanical Turk such that only those who have completed at least 50 MTurk HiTs with over 95% approval rating were able to participate. All experimental procedures described hereafter were carried out in accordance with the guidelines of the University of California, Irvine and University of California, Riverside, Institutional Review Boards (IRBs). Ethical approval for these studies was obtained from the University of California, Irvine and University of California, Riverside IRBs, and informed consent was obtained from all the study participants according to disclosures approved by the University of California, Irvine and University of California, Riverside IRBs. Regardless of collection modality, all participant data underwent rigorous quality control by independent raters blind to the study hypotheses. Raters were instructed to identify individuals whose data indicated they were inattentive to the experiment or appeared to be automating responses for more than one quarter of the trials. Raters examined reaction times and response patterns for each participant and coded each participant as either 0—do not exclude, 1—consider excluding, or 2—definitely exclude. Raters also provided reasonings for exclusion. Some reasons for exclusion included: high frequency of no-response trials, unrealistically low or high reaction times (which may have been indicative of connectivity or computer issues or automated respondents—e.g. bots or scripts), “button-mashing” (pressing the same button/choice for the majority of the experiment), or not sampling all three choices throughout the task (for instance, alternating between only two choice options for the majority of the task). Ratings from two independent raters were summed, and participants with scores greater than two were excluded. In other words, we excluded participants whom at least one rater identified as ‘Definitely’ excluded (Score 2) and the other rater scored at least ‘Maybe’ excluded (Score 1 or 2). Of the 451 participants originally recruited across both experiments, 83 participants were excluded on this basis.

We also excluded participants for whom the learning model did not fit better than chance (p(choose) = 1/3 for all trials), as the parameters for these participants would not distinguish from a participant who made random choices. An additional seven participants were excluded this way (all from Experiment 2), which left 361 participants (across Experiments 1 and 2) for our analyses: 226 participants were retained in Experiment 1 (112 online/114 in-person, 88 male/137 female/1 other, ages 18–77, mean (sd) age = 38.6 (18.8)), and 135 participants were retained in Experiment 2 (117 online/18 in-person, 66 male/68 female/1 other, ages 18–74, mean (sd) age = 40.6 (16.2)). Participants had to complete a brief tutorial and screener of the three-armed bandit task to be eligible for the full experiment. The screener consisted of an interactive tutorial of the task and practice trials of the learning phase to ensure participants understood the instructions and were paying attention to each trial. Individuals had two opportunities to complete the tutorial and 10 practice trials with a minimum 70% accuracy to continue on to the full experiment. Those who were ineligible to continue on to the main task were compensated $2 for the time spent on the tutorial. Participants received monetary compensation for their participation: $12 for completing the experiment, and an additional bonus of up to $8 obtained throughout the task.

Participants were recruited for two separate sessions to complete their two tasks (one of the choice tasks and the Mnemonic Similarity Task). During early phases of data collection, the order of recruitment for each task (choice task and MST) were counterbalanced in case there may be differences in retention as a function of which task was performed first. Our pilot data revealed that roughly 5 out of every 8 participants generally returned for a second session, regardless of which experiment was performed first. For participants who were recruited first through the choice experiment, participants who completed the entire experiment were invited back to complete the Mnemonic Similarity Task (MST)^27^. For other participants, those recruited to complete the Mnemonic Similarity Task first and those who completed the MST were invited back to complete one of the three-armed bandit tasks. Ultimately, 130 participants from Experiment 1 (48 male/82 female/1 other, ages 18–77, mean (sd) age = 37.0 (20.5)) and 97 participants from Experiment 2 (45 male/52 female, ages 18–73, mean (sd) age = 40.3 (16.7)) completed both the MST and three-armed bandit tasks. Participants received a fixed fee of between $3 and $5 for their participation on the MST, depending on the experiment modality and date of data collection (during the course of data collection we adjusted our per-hour participant compensation to account for inflation).

To examine the possibility that there may be significant differences between our online and in-person participant samples influencing our main analyses, we examined the distribution of each model parameter of interest (ꞵ_sampler_, ꞵ_TD_, ꞵ_perservation_) for comparable online and in-person participants (younger adults between the ages of 18–40). For each parameter, we conducted an independent samples t-test to verify that the parameter distribution did not differ between online and in-person samples. All comparisons showed there was no significant difference in parameter distributions as a function of online or in-person data collection (p > 0.2 for all 3 t-tests), alleviating possible concerns that data collection source may be significantly influencing our variables of interest.

### Three-armed bandit task

#### Tutorial/practice

Participants first completed a practice tutorial screener to determine eligibility for the main task. Participants engaged in an interactive tutorial during which they practiced the various aspects of the task: (1) pressing keys corresponding to each card deck option of the three-armed bandit task in a “virtual casino”, (2) experiencing the probabilistic payoff structure of each option, (3) making choices and remembering the casino room in which various item rewards (“lottery tickets”) were received within the trial’s time window, and (4) practicing the memory probe recognition trials in Phase 2. The instructions unfolded slowly in order to give participants adequate opportunities to familiarize themselves with all aspects of the task, and there was a final quiz. If participants did not successfully complete the quiz with at least 70% accuracy, they were given the opportunity to repeat the tutorial and quiz. Those who failed a second time were ineligible to continue on to the main task.

The main task followed protocols outlined in the method section of previous work that used this three-armed bandit task^31^. Additional information on the task can be found there.

#### Phase 1 (learning phase)

**Figure 1:**
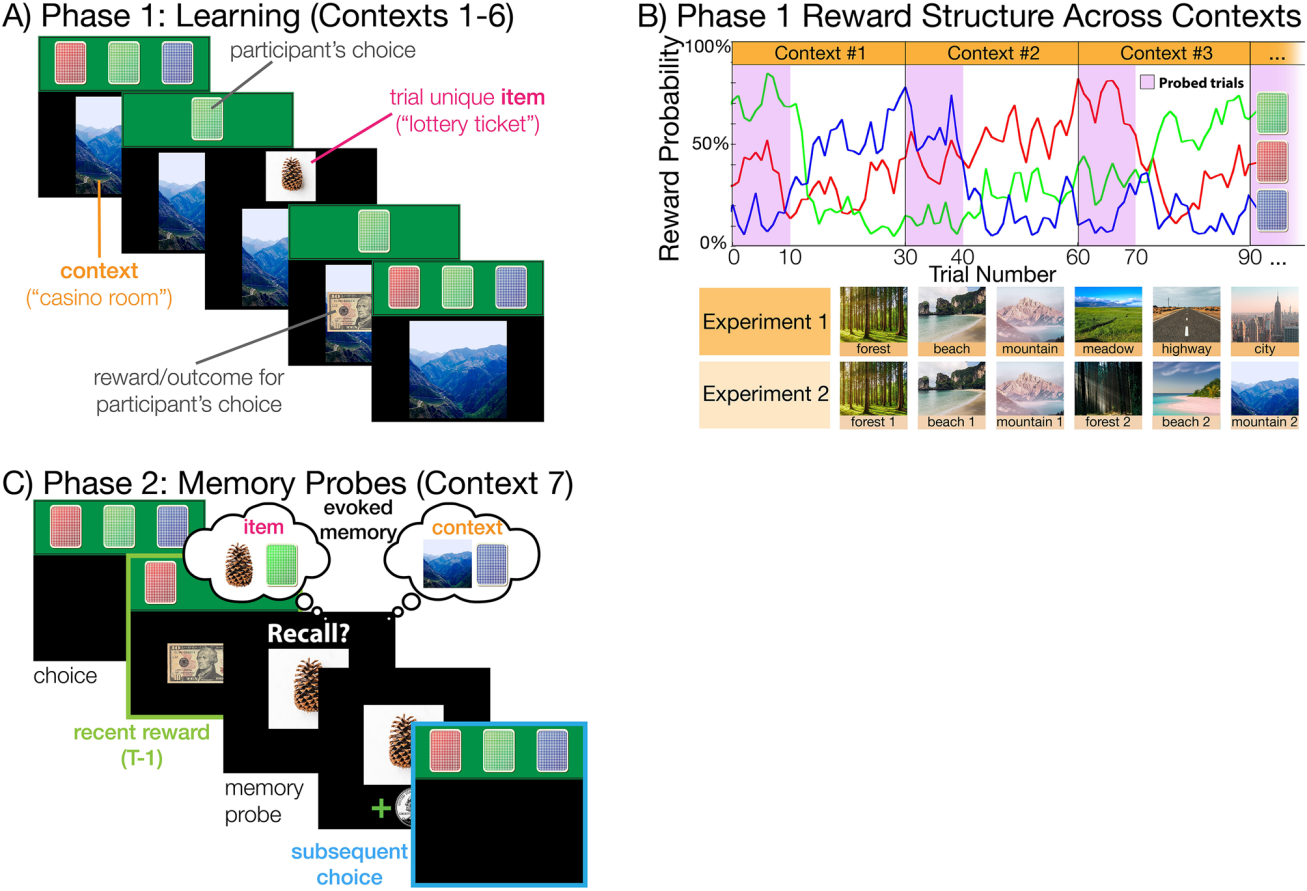
Three-armed restless bandit task. Across two experiments, participants performed a three-choice decision-making task with uncertain reward contingencies and trial-unique memoranda. Task was adapted from Bornstein and Norman (2017)^31^. **(A)** Learning phase. Participants first performed 180 choice trials, split into six consecutive “casino rooms.” Each “room” lasted for 30 trials and consisted of three differently-colored card decks on a green background at the top of the screen, and a scene image at the bottom of the screen. The scene remained on screen for all 30 trials of each room. After selecting a card deck, the top card was turned over to reveal a trial-unique item photograph. Then, a reward amount—either a picture of a US $10 bill, or a phase-scrambled version of the same, the latter indicating a $0 reward for that trial. Participants were told to treat each trial as equally important, and that their total payout would depend on the choice made on one randomly-selected trial. They were also told that the decks and “dealer” would follow them through each room of the casino. **(B)** Reward structure. Each card deck had a different, steadily-varying, probability of paying out a reward. Decks were initialized to 60%, 30%, and 10% randomly for each participant—these payouts then varied across trials according to a gaussian random walk tending towards one of those three probabilities as its center. After trial 10 in each room, the centers were rotated such that the new highest-paying deck was different from the previously highest-paying deck. The payout rankings continued uninterrupted into the next room, reinforced by the payout of the highest deck being set to 100% for the first three trials of that room. Though payout order did not change between rooms, context images did. In Experiment 1, each room had a highly distinct image as a background. In Experiment 2, two rooms were *perceptually aliased*, with background images that were highly visually similar (i.e. two forests, two beaches, two mountains). Critically, the payouts for the aliased rooms were opposed to each other—the highest paying deck in the first room of each kind was different from the highest paying deck in the second. **(C)** Memory probe phase. Participants were told that the remaining 120 trials would be performed in an “unfinished” room of the casino, which had no background image. The payouts continued to drift slowly and rotate every 30 trials, however there were no further room boundaries. Here, choices no longer resulted in an item image—only a reward outcome. Interspersed among the 120 choice trials were 60 *memory probe trials*, 50 of which presented an image previously observed, and 10 of which presented a novel image. The key measure of interest was the degree to which participant choices on trials following the memory probe were biased towards choices made in the room brought to mind by the probed image.

Participants completed 180 choice trials of a 3-armed bandit task that occurred across 6 different contexts (“casino rooms”; Fig. 1A). Participants were told they are playing a card game across 6 casino rooms and that they should try to maximize reward by picking one of three card decks that most often leads to reward. The context was presented as a background image on the screen, which changed every 30 trials. In Experiment 1, the contexts presented in Phase 1 consisted of 6 unique outdoor scenes (Fig. 1B, “Experiment 1”). In Experiment 2, there were 2 contexts for each of 3 outdoor scene categories (i.e., 2 beach, 2 mountain, 2 forest). One context from each category was sampled first (e.g., beach #1, forest #1, mountain #1) before sampling from the same-context foils (e.g., forest #2, mountain #2, beach #2). There was an additional constraint that two rooms from the same category (e.g., forest #1 and forest #2) were not assigned to adjacent contexts (i.e., context 3 and 4).

Participants were told that the deck that most often leads to reward can change over time, so the optimal strategy is to periodically sample all choices to ensure that they are picking the best deck. Critically, it was emphasized that the decks “and dealer” followed participants between rooms—this feature was emphasized by the actual payout probabilities, described below. Participants had up to 2.5 s to make their choice by using the “1”, “2”, or “3” key on their keyboard to select one of the three presented card decks. After making their choice, participants were shown the chosen deck in isolation for 0.5 s, followed by a trial unique object image for 2 s, followed by a $1 or $0 reward for 1.5 s (Fig. 1A). Each trial was tagged with a trial-unique object image to later be used as an item-memory probe and remind participants of rewards received on a given trial. Participants were told that the trial-unique objects are “lottery tickets” that they will have to recognize to receive their associated rewards later. Participants learn to follow which deck most frequently yields reward through trial-and-error, while simultaneously learning to associate the different contexts (casino rooms) with trial-unique items (lottery tickets) experienced across the 180 trials.

#### Phase 2 (probe phase)

During the probe phase, participants completed an additional 120 choice trials of the 3-armed bandit task. However, during this phase, no context or trial-unique item was presented in any of the trials (Fig. 1C). Instead, 60 memory probe trials were pseudorandomly interspersed amongst the choice trials. During a memory probe trial, participants were shown an object from Phase 1 to serve as a reminder of its associated reward and context from the learning phase. Participants were instructed to continue to make choices in this “unfinished” room and follow the deck most frequently associated with reward. They were also instructed that they may occasionally be shown an object image and asked to recognize if it were one of the lottery tickets they collected from Phase 1. Participants had up to 3 s to determine if the object was previously seen. Each correctly identified lottery ticket added a small monetary bonus (+ $0.05), and each incorrectly identified ticket resulted in a small monetary deduction (−$0.05).

#### Reward structure

In Phase 1 and 2, each card deck followed a probabilistic reward structure that changed throughout the task (Fig. 1B). Each deck was assigned to initial payout probabilities ($$\pi$$, the odds the deck would return a $10 reward, rather than $0) of 60%, 30%, or 10% randomly, without replacement. These payout probabilities then slowly drifted across trials (*t*) within each context according to a decaying Gaussian random walk with reflecting bounds at 5% and 95% that was centered at the target probability ($${\theta }_{i}$$) assigned to each deck (*i*)^31^:

1 $${\pi }_{i,t+1}=\lambda {\pi }_{i,t}+(1-\lambda ){\theta }_{i} + \nu$$

Parameters were set as follows: $\lambda$ (stickiness) was 0.6, $\nu$ (diffusion noise) was a zero-mean Gaussian with SD 8. To confirm to participants that the decks “carried with them” across rooms, the stickiness parameter was temporarily set to 0.95 for the first three trials of each room.

The key feature of the payoff structure was as follows: the target payout probabilities ${\theta }_{i}$ were reassigned after the first 10 trials within a given context and persisted for 30 trials before resetting again. Critically, item probes were selected only from these first ten trials, resulting in the memory probes having a reward distribution that is dissociable from the reward distribution of the context within which the item was presented. For both experiments, four payoff time series were pre-generated according to this procedure and randomly assigned to participants in the task. In Experiment 2, there was an additional constraint that the reward distribution for one context (e.g., forest #1) was distinct from its same-category foil (e.g., forest #2), thereby allowing us to determine if evoked memory content from memory probes in Phase 2 is consistent with memory for 1) the target context, 2) the same-category lure, or 3) a gist representation of the category (target + lure).

## Analysis of choice behavior

### Regression analysis.

Our initial analysis of interest examined how reminder probes—and the trials and contexts they referred to—affected choices in Phase 2. Following our previously employed procedure^31^, we constructed a three-part logistic regression model, with each part corresponding to one of the three card decks. For each deck and each trial, we modeled: for all trials, the identity of the deck chosen on the previous trial (*DI*: 1 for the given deck, 0 for others) and the recent rewards up to three steps back (*DR*: 1 if the given deck was chosen and rewarded on trial t − *k*, k = 1..3, 0 otherwise); for trials immediately following the memory probes, the deck identity (*EI*: 1 if the same deck as probed, 0 otherwise), the deck-specific value received (*ER*: 1 if the same deck and rewarded, 0 otherwise) on the individual trial reminded by a memory probe, and the average reward of the probed deck, across the reminded context room (*EC*; Eq. 2).

2 $$E{{C}^{C,i}} = \frac{\# \, of \, choices\, of\, i \, resulting\, in\, \$10 - \# \, of\, choices\, of\, i \, resulting\, in\, \$0}{\# \, of \, times\, i\, chosen\, in \, C}$$

This quantity, *evoked context reward* (*EC*^*C,i*^) reflects the expected value of samples of this deck *i* from the reminded room context *C*. The total regression model was thus:

3 $${C}_{i,t} \approx {{\beta }^{D{I}_{i}}D{I}_{i,t-1}}+{\sum }_{k=1}^{3}{\beta }^{D{R}_{i}}D{R}_{i,t-k} +{{\beta }^{E{I}_{i}}E{I}_{i,t-1}+{{\beta }^{E{R}_{i}}E{R}_{i,t-1}}+ {{\beta }^{E{C}_{i}}E{C}_{i,t-1}}}$$

where *C*_*i,t*_, the dependent variable, reflects whether deck *i* was chosen on trial *t* (1 or 0).

The resulting design matrix thus had seven columns, and 360 rows—one for each deck and choice trial combination. For the second experiment, a second analysis was run which included an eighth column representing the “gist-level” evoked context reward computed across the trials from the combined target evoked context and its paired *lure* context room. This regressor was orthogonalized against the target context regressor using Gram-Schmidt orthonormalization as implemented by the SPM8 function spm_orth^34^. The coefficients produced by all regressions were tested against zero across the population by two-tailed t-test.

#### Computational model

The *hybrid model* consisted of a participant-specific mixture of two value-estimation processes, each drawing on previous experience in different ways^16,31^. The first process, *reinforcement learning*, is an incremental, error-driven updating process which learns an expected value, *V*_*t,RL*_*(x)* for each card deck, *x*, at each timepoint *t* (Eq. 4;^35^). This process had one free parameter, the learning rate (${\alpha }_{RL})$, which was fit to each participant individually, and captured the degree to which the expected value was updated by the difference between the reward received for the choice made on that trial (*R*_*t*_*(x)*) and the value to be expected on the basis of previous experience (*V*_*RL,t*_*(x)*):

4 $${V}_{RL,t}(x) = {V}_{RL,t-1}(x) + {\alpha }_{RL}[{{{R}_{t}\left(x\right)}- V}_{RL,t-1}(x)]$$

Choice values were initialized to zero, and were not updated for options not chosen on a trial.

The second process, *memory sampling*, estimated values on the basis of a single “sample” drawn from past experiences with each choice option. The probability of a particular past experience serving as the value was proportional to how recently in the past it was selected, with recency weighted according to the decay parameter (${\alpha }_{sample})$:

5 $$P({V}_{sample}(x)=={R}_{i}) = {\alpha }_{sample}(1-{\alpha }_{sample}{{{)}^{t-i}}}$$

For the model-fitting procedure, the value was computed as the expectation-weighted average value across all combinations of possible samples for each choice option^16^.

These values were transformed into action probabilities *P*_*t*_*(x)* via a *softmax* action-selection function, with separate *inverse temperature* parameters (${\beta }_{sample}, {\beta }_{RL})$ reflecting how sensitive action selection is to the value differences estimated by that process, for that participant. A third parameter (${\beta }_{p})$ reflected the influence of perseveration, or “stickiness” of recent choices, independent of the estimated action values.

6 $${P}_{t}(choose x) = \frac{exp{{[\beta }_{p}I({x}_{t}=={x}_{t-1}) + \beta }_{sample}{V}_{sample,t}(x) + {\beta }_{RL}{V}_{RL,t}(x)]}{exp{{[{\sum }_{x=1}^{3}{\beta }_{p}}I({x}_{t}=={x}_{t-1}) + {\beta }_{sample}}{V}_{sample,t}({x}_{t}) + {\beta }_{RL}{{V}_{RL,t}}({x}_{t})]}$$

The resulting temperature parameters were treated as the variables of interest for our analyses below.

Models were fit using maximum a-posteriori estimation via unconstrained optimization (MATLAB function fminunc). Parameters were input to the optimizer as unbounded real numbers, and then logistic-transformed within the likelihood function to appropriate bounds: ${\alpha }_{sample},{\alpha }_{RL}\sim [\mathrm{0,1}]; {\beta }_{sample},{\beta }_{RL}\sim [\mathrm{0,20}]; {\beta }_{p}\sim [-\mathrm{3,3}]$. Randomly-sampled starting points were selected for each run of the optimizer until the minimum observed value did not change for five consecutive runs.

The following weakly informative priors, truncated within the ranges specified above, were used in the final likelihood calculation: ${\alpha }_{sample},{\alpha }_{RL}\sim Beta(\mathrm{1.1,1.1}); {\beta }_{sample},{\beta }_{RL}\sim Normal(0, 10)T[\mathrm{0,20}]; {\beta }_{p}\sim Normal(\mathrm{0,10})T[-\mathrm{3,3}].$.

### Mnemonic similarity task

**Figure 2:**
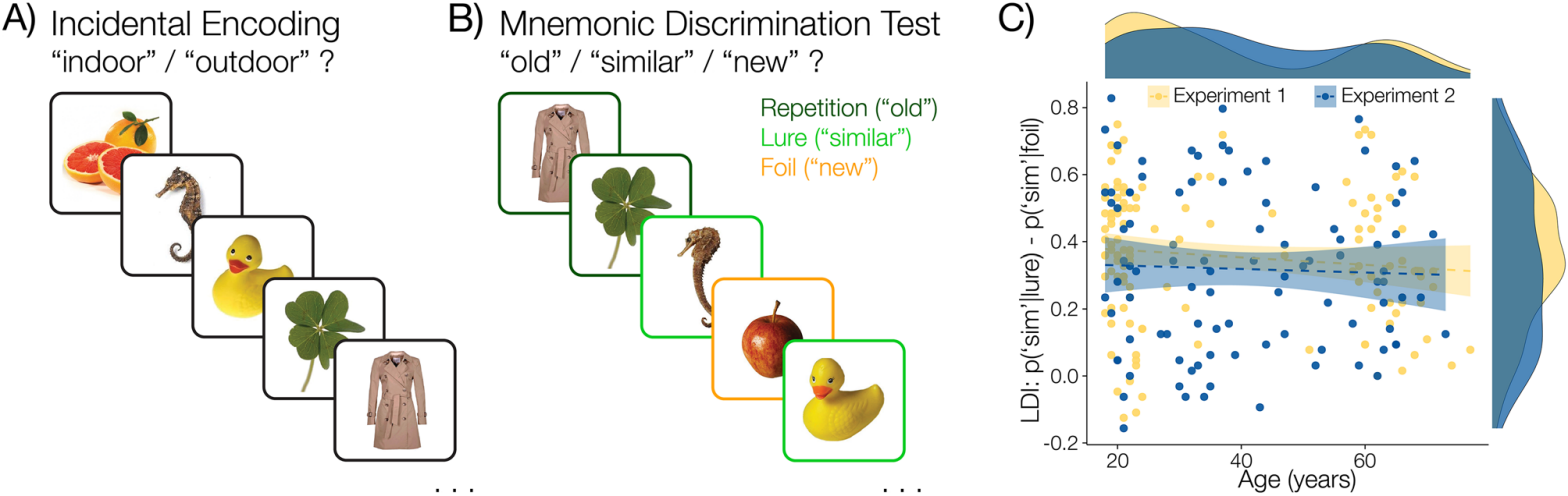
Mnemonic similarity task. **(A)** Participants view a sequence of objects during an incidental encoding phase in which participants classify each object as an indoor or outdoor object. **(B)** In a surprise discrimination test, participants view a series of objects and are asked to determine whether each object is “old,” “new,” or “similar” relative to the items that were shown during the encoding phase. **(C)** Correlation between age and lure discrimination scores (LDI) for each experiment. Shaded bands indicate 95% confidence intervals around the trendline.

Participants were invited to return to complete the Mnemonic Similarity Task^27^ in a separate session (Fig. 2). The Mnemonic Similarity Task was used as an individual difference measure of memory precision for each participant, which we reasoned would play a role in the degree to which participants would be influenced by evoked memories following probe trials in the second phase of the choice task. In the study phase, participants view 128 object images in an incidental encoding task in which participants are tasked with judging whether each image is an indoor or outdoor object. In a surprise recognition test, participants are shown 192 object images, with one-third consisting of exact repetitions of the same object that was shown during study (Fig. 2B, “repetition”), one-third consisting of perceptually similar objects (Fig. 2B, “lure”), and one-third consisting of objects that were never shown during the study phase (Fig. 2B, “foil”). Participants are asked to judge whether each object is old, similar, or new relative to what was shown during the study phase. A lure discrimination index (LDI) was computed for each participant using the following formula: LDI = p(“similar”|Lure) − p(“similar”|Foil). The LDI is widely used to measure individual and age-related differences in mnemonic discrimination, with higher scores being associated with better memory precision^27,28,36–39^.

### Parameter correlations

The critical test of our hypotheses involved correlations between parameters extracted from computational model fits, described above, and external measures of interest (age, LDI). Because the hypothesized relationship between model parameters and these measures is ordinal, and because the variables exhibit “ties”, we report the rank-based Kendall’s τ_b_ correlation statistic^40^. Comparisons between correlations (i.e. across experiment populations) were assessed by z-test after first converting to the corresponding linear *R* value using the mapping $R=sin(0.5*\pi *\tau )$^41^.

To determine the specificity of LDI ~ parameter relationship, correlations were also examined after regressing out the linear relationship with age. This computation was performed by first estimating the residuals of the regression model *X* ~ $\beta$*age, and performing the corresponding correlation against the resulting values.

## Results

### Experiment 1

**Figure 3:**
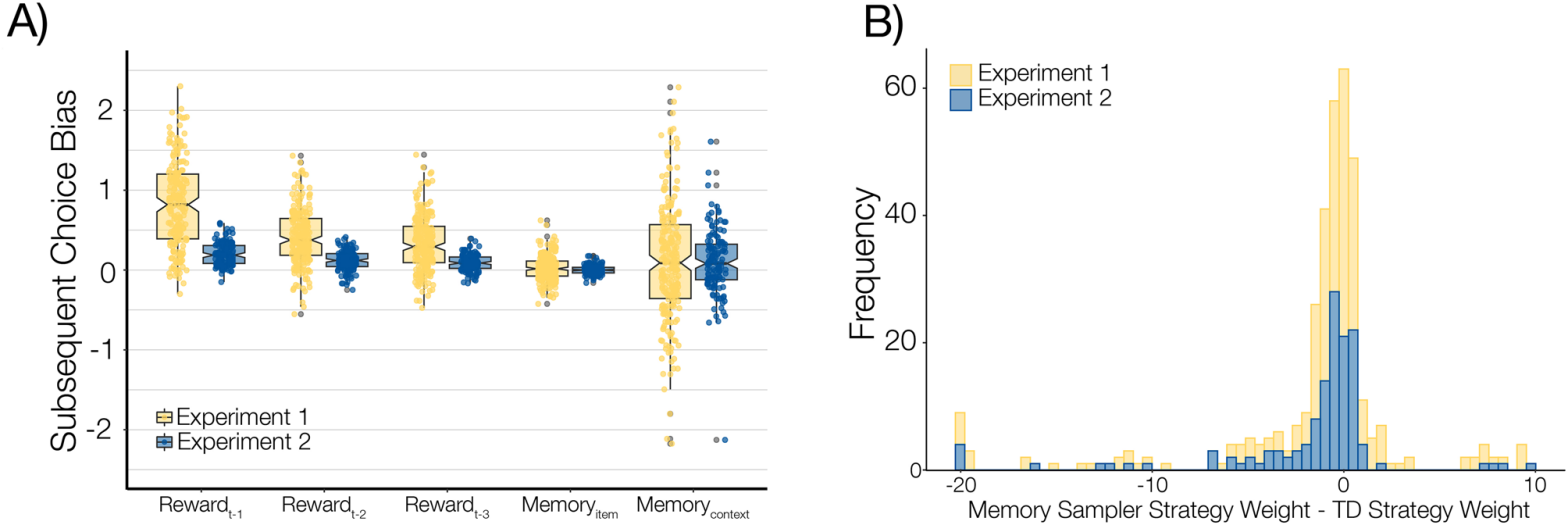
Influence of recent reinforcement and memory on choices. (**A**) Recent reinforcement and reminded memories both influence choices. Boxplots of logistic regression beta estimates (log relative choice odds) quantifying the contributions of reinforcement learning (recent reward history) and memory content (specific item memory vs. context memory) on subsequent choices following memory probes in Phase 2. In both Experiments, we observed a significant and consistent influence of both recent rewards and memory probes on subsequent choices (experiment 1: yellow; experiment 2: blue). (**B**) Subjects exhibit a mixture of memory sampling and temporal-difference reinforcement learning strategies. Across participants, the relative use of temporal-difference reinforcement learning and memory sampling varied. The bulk of subjects exhibited a near-equal mixture of the two, but overall recent reinforcement was a greater influence on choices (Experiment 1, yellow: $t(225)=-3.43,p<.001$; Experiment 2, blue: $t(134)=-3.75, p<.001$).

*The influence of memory intrusions on choice is observable across the lifespan.* Following the preceding study which examined behavior in this task in young adults (Fig. 1^31^), our measure of interest was performance on choice trials following recognition memory probes. Previous research using this decision-making task has shown that memory probes result in intrusive recollection of past choices such that evoked item memory content and evoked context memory content have separable contributions to subsequent choice behaviors, independently of recently received rewards^31^. Our initial question of interest was whether this general pattern of findings was replicable in our lifespan sample of participants. Following previous work, we ran a multiple regression to model the contributions of each of our variables of interest on choice behavior. As previously observed in young adults^16,31^, there was a significant influence of both the reminded trial and reminded context on choices following the memory probe, while controlling for the influence of recent rewards (Fig. 3A; $item: t(226)=2.09, p=0.038; ctx: t(226)=2.32, p=0.021$).

Previous work has documented a decline in memory specificity with age^42^. Such an effect could dampen the intrusive influence of retrieved values on decisions. On the other hand, other work has shown that memory’s effect on value-guided choice reflects an elaborated retrieval process, and so the presence of memory-biased choice would itself necessitate the retrieval of specific information^43^. Therefore, we examined whether memory effects were modulated by either chronological age or cognitive decline, the latter indexed by the Lure Discrimination Index (LDI) computed on performance during the separately-administered Mnemonic Similarity Task (Fig. 2). The LDI, which measures the participants’ selective accuracy in identifying similar lures, is widely used as a behavioral measure of the specificity of encoded memories, and is known to decrease with age and more specifically with the onset of age-related cognitive decline^28^. Intriguingly, and consistent with the idea that value itself is the specific memory content of interest in this task^43^, we found that neither age nor LDI modulated the effect of item or context-based intrusions ($age*item: {r}_{\tau }(225)=-0.039, p=0.391 LDI*item: {r}_{\tau }(128)=-0.925, p=0.355$; $age*ctx: {r}_{\tau }(225)=0.050, p=0.271; LDI*ctx: {r}_{\tau }(128)=-0.583, p=0.560$).

#### Memory sampling and RL are separately modulated by memory precision and age

**Figure 4:**
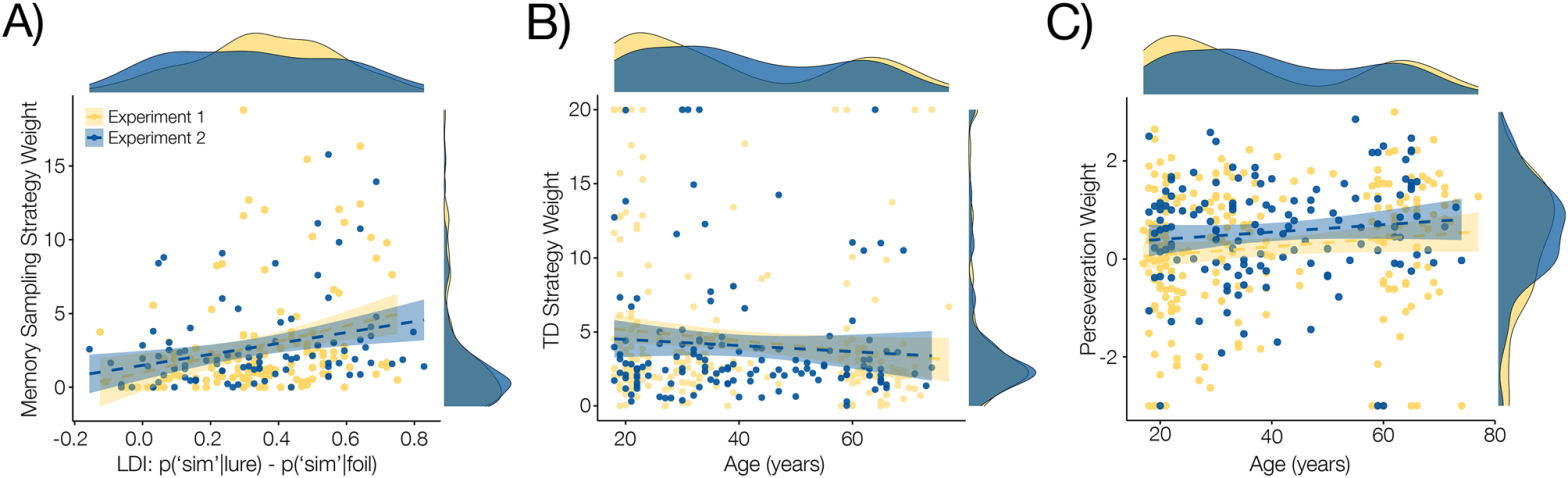
Memory precision and age differentially modulate the influence of experience on decisions. Both Lure Discrimination Index and Age separately tracked individuals’ use of distinct experience-derived choice influences. **(A)** Memory sampling increases with memory precision. Consistent with theoretical and empirical findings that uncertainty is a critical factor in the weight of decision strategies, increased memory precision was associated with an increase in the use of the memory sampling strategy in choice: ${r}_{\tau }(225)=.228, p<.001$. **(B)** Choices are less sensitive to reinforcement learning with age. Consistent with previous findings that choice noise increases with age, participants exhibited less influence of Reinforcement Learning-derived values with age. ${r}_{\tau }(359)=-.087, p=.015$. (**C)** Perseveration increases across the lifespan. Consistent with previous findings, participants exhibited a greater tendency towards perseverative responding with age. ${r}_{\tau }(359)=.101, p=.0048$. All correlations plotted for each experiment individually (Experiment 1: yellow, Experiment 2: blue, shaded bands represent 95%CI around the trendline), and statistics reported for the combined sample. Correlations were not different between experiment samples ($LDI*{\beta }_{sampler}: z=-0.21, p=.417; Age*{\beta }_{TD}: z=-0.704, p=.241; Age*{\beta }_{persev}: z=0.446, p=.328$).

Having observed that memory intrusions were consistent across the lifespan and the range of memory precision scores, we next examined factors that guided the use of behavioral strategies to perform the choice task. To answer this question, we fit to choice behavior a hybrid computational model implementing two distinct approaches to value-based decision-making. The key measure of interest in this model is the degree to which an individual’s choices reflect values estimated by *memory sampling*^15,16^ and also values estimated using standard temporal-difference reinforcement learning^44^. Models were fit to choices in the first six rooms of the “casino”, absent the influence of memory probes. The result of this model fitting is that the reliance on each strategy is reflected in the inverse softmax temperature parameters that best describe each participant’s behavior as a function of the values estimated by each process. Overall, we found that most subjects exhibited a near-equal mixture of TD and memory sampling, but that TD was a greater contributor to behavior across the population ($t(225)=-3.43,p<.001$; Fig. 3B). Consistent with the hypothesis that the use of memory-based decision strategies is guided by the relative uncertainty of memory representations^12,19–21^, we found that, at a subject-level, increased memory precision (as measured by LDI) predicted the use of a memory sampling strategy (${r}_{\tau }(128) = .22, p < .001$); this effect remained even when regressing out age (${r}_{\tau }(128)=.13, p=.03$). Notably, LDI did not predict the use of the reinforcement learning strategy ($LDI*{\beta }_{TD}: {r}_{\tau }(128)=.01, p=.9$), but, consistent with previous work suggesting increased choice noise and perseveration with age^45^, we found that age predicted less reliance on reinforcement learning (${r}_{\tau }(128) = -.11, p = .019$; Fig. 4A), as well as a greater influence of perseveration (${r}_{\tau }(128) = .11, p = .017$; Fig. 4B).

### Experiment 2

#### Age-related memory decline predicts the intrusion of gist, rather than specific-context memories on choice

Experiment 1 demonstrated that the effect of memory intrusions on choice was not a function of age or memory specificity, but that both factors contributed to the relative influence of decision strategies on choice. We thus conducted a follow-up experiment examining whether a more fine-grained distinction, about the *content* of intrusive memories, might be revealed by a direct test of memory specificity. Specifically, motivated by previous research suggesting that age-related memory decline is associated with a greater reliance on ‘gist’ representations, rather than specific memories^46–49^, we examined whether the content of probe-triggered memories was more ‘gist’-like in individuals with lower memory precision. In our previous study using this task^31^, we found that neuroimaging measures of specific-scene reinstatement on each trial–which indicated the degree to which an individual reinstated alternative, rather than target, contexts on that trial–modulated the value that guided subsequent choice. This finding is consistent with the idea that samples are drawn from the context reinstated at the time of each decision. Therefore, we reasoned that making the distinction between scene contexts more difficult might lead individuals with lower memory precision to sample, at least partly, from the alternate, ‘lure’ room.

**Figure 5:**
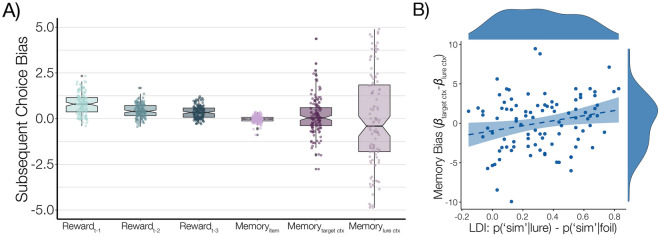
Experiment 2: two kinds of reminded context influence choices. (A) Specific and gist context memory compete for influence on choice. Boxplots of logistic regression beta estimates measuring the contributions of reinforcement learning (recent reward history) and memory content (specific item memory, evoked target context memory, and evoked lure context memory) on subsequent choices following memory probes in Phase 2. Target and Lure contexts compete for variance in this analysis, reducing the effect of context observed in Fig. 3 (blue). **(B)** Memory precision tracks the influence of specific and Lure context memory. Across subjects, individual estimates of memory precision (LDI) predict a greater influence of memory intrusions for the target reminded context relative to its same-category lure (${r}_{\tau }(95)=.148, p=.0329$).

In Experiment 2, individuals again performed a three-armed restless bandit task across six distinct room “contexts.” However, in this experiment the contexts were split into perceptually aliased pairs—two scene images each of mountains, beaches, or forests (Fig. 1B). Critically, we controlled the payoff probabilities such that the best-performing card deck in the first room of a given type was not the best-performing in the second room. This allowed us to distinguish the effect of specific-context intrusions (e.g. ‘this particular beach’) versus intrusions based on a more ‘gist’-like representation (e.g. ‘beaches’). We first analyzed whether, consistent with Experiment 1 and previous observations, behavior was significantly modulated by reinstated target context intrusions, across the lifespan. Indeed, across the population, participants showed an effect of context-memory intrusions ($t(133)=2.81, p=0.006$). We next examined whether age or LDI correlated with the influence of the target context relative to its same-category lure (the other image of the same type (‘beaches’)). As expected, including the (orthogonalized, see *Methods*) lure context reward in the regression competed with variance for the target context, reducing the effect of the former (Fig. 5A), suggesting that, across the population, participants relied on a mixture of both target and lure context representations (i.e., a ‘gist’ category level representation of the context). We then examined the relative reliance on one (target context) versus the other (lure context), at a participant level, as a function of age and LDI (Fig. 5B). Consistent with the idea that age-related cognitive decline in memory precision alters decision-relevant representations, we found that the influence of the target context on choice was correlated with LDI score (${LDI*Target-Lure: r}_{\tau }(95)=0.148, p=0.0329)$, but not age ($Age*Target-Lure: {r}_{\tau }(95)=0.070, p=0.317$; difference between correlations: $z=0.854, p=0.197$); the relationship between LDI and target context influence held even after controlling for age (${r}_{\tau }(95)=.144, p=.038$).

#### Strategy use

 Again, participants placed greater weight on the temporal-difference learning strategy than the memory sampling strategy ($t(134)=-3.75, p<.001$; Fig. 3B), and memory precision, but not age, predicted the use of the memory sampling strategy ($LDI*{\beta }_{sampler}: {r}_{\tau }(95)=.238, p<.001; age*{\beta }_{sampler}: {r}_{\tau }(133)=-.027, p=.641; z=3.149, p=.001$). In this smaller sample, neither the correlation between age and TD strategy ($Age*{\beta }_{TD}:{ r}_{\tau }(133)=-.0582, p=.321$; Fig. 4B, blue) nor between age and perseveration ($Age*{\beta }_{persev}: {r}_{\tau }(133)=.0766, p=.192$; Fig. 4C, blue) was statistically significant. Further, the differences between these correlations and those observed in Experiment 1 were not themselves significant ($Age*{\beta }_{TD}: z=-0.704, p=.241; Age*{\beta }_{persev}: z=0.446, p=.328$)^50^. Combining across experiments, both effects of age on strategy use were statistically significant ($Age*{\beta }_{TD}: {r}_{\tau }(359)=-.087, p=.015; Age*{\beta }_{persev}: {r}_{\tau }(359)=.101, p=.0048$; Fig. 4B, C).

## Discussion

Individuals across the lifespan are often called on to make value-based decisions under uncertainty that have important consequences for themselves and others. However, despite extensive characterization of age-related differences in decision-making capacities, little is known about the mechanisms by which these arise. Here, we examined the hypothesis that age-related differences in decisions may be linked to age-related differences in memory, which have been studied for longer and are better understood at a mechanistic level.

We specifically focused on the role of *memory sampling*, a process of retrieving memories of previous choice instances during decision deliberation that has been shown to affect neural signals and behavioral outcomes^13,15,16,31,51^, even during tasks for which it is maladaptive to rely on episodic memories^16,31,52,53^. We used a previously-validated task that, in younger adults, identified separable influences on choice of values associated with *item* and *context* memory, the latter corresponding with neural markers of context reinstatement that linked it to specific retrieved experiences—even if those experiences were incorrectly attributed^31^. In Experiment 1, we observed that a lifespan sample of participants replicated previous findings obtained in young adults. Further, we extended the previous findings by showing that *memory precision*—as measured by the separately-administered Mnemonic Similarity Task^27^—modulated the influence of memory sampling, with greater precision leading to a greater effect of memory sampling on choice. In parallel, and in keeping with previous findings in similar repeated decision-making tasks, chronological age was associated with increasing noisiness of choices relative to values estimated using standard reinforcement learning, and a concurrent increase in perseverative responding. In Experiment 2, we delved further into the relationship between memory precision and choice, by identifying a role for memory precision in selecting which memories are sampled. Specifically, we designed a variant of the previous task in which sampled context memories could be identified as specific or ‘gist’-level (e.g. ‘beaches’ as opposed to ‘that one particular beach’), with each having distinct, opposing effects on choice. We found that lower memory precision was associated with a greater reliance on gist-based memory during memory sampling. This result concords with an extensive literature on older adults’ greater reliance on gist, rather than specific, memories^48^, and connects it to a recently developed computational model of action selection to explain why some memories are sampled during decision deliberation, rather than others^22^.

These findings suggest that age-related differences in decision-making result at least in part from interactions between systems for memory-guided and reinforcement learning-based action selection. More specifically, they link age-related differences in specific memory functions, in particular pattern separation, to age-related alterations in value-based decision-making. The finding that older adults, broadly, may be less sensitive to values estimated using reinforcement learning suggests that this decline in sensitivity could be at least partly mitigated by a greater reliance on recency-weighted memory sampling, if memory precision is spared. Examining the interactive influence of these systems on choice across the lifespan is a promising topic for future research. For instance, these results suggest that interventions that aim to improve older adults’ sensitivity to value may profitably focus on improving access to, or encoding of, specific choice-related memories that may be of critical importance to future decisions.

Our results also bear on previous findings regarding the nature of mnemonic discrimination deficits in aging. There is some evidence that age effects on discrimination are amplified for objects compared to scenes^54,55^. On the one hand, this suggests that our scene-based context manipulation in Experiment 2 was appropriate for participants across the lifespan and may explain why age did not modulate the effect of context-based intrusions on choices. On the other hand, however, our use of a separate object-based discrimination task to assess memory precision may not capture the same pattern separation processes taxed by the context-specific value-based decision task. Future examinations of the influence of age and memory precision (LDI) may benefit from using scene, not just object, mnemonic discrimination tasks.

More broadly, the finding that memory precision guides decision strategy has implications for the study of the computational nature of goal-directed decision-making more broadly^56^. For one, that memory sampling and RL appear to be independently modulated by memory precision and age supports suggestions that these systems may indeed be distinct approaches to action selection^17,32^, with the former supporting both model-free^16^ as well as model-based^6^ choice. Second, the finding that memory precision indexes both the specificity of contexts for sampling and also the overall reliance on memory suggests that uncertainty is computed dynamically and adjusted for in response to the available momentary evidence^20–22^, rather than cached as a controller-specific quantity dictating patterns of choice across trials^57^. At a neural level, future studies could investigate whether the seemingly independent differences in RL-based and memory-based choice processes are related to differences in fronto-striatal and medial temporal systems, respectively, extending prior work that typically examines these brain-behavior relationships separately for each neural system^58^.

This study is not without its limitations: most of the limitations come from potential issues with online data collection. Because this project began during a global pandemic, a large majority of our data was collected online. As a result, our data was collected in a largely unsupervised fashion, and thus there is a risk that the data quality may not be as good as data collected through in-person means. We have taken several steps to remedy this such as: 1) setting eligibility filters to ensure that we had reliable online participants who have good experience participating in online studies and surveys, 2) comparing model parameter distributions between in-person and online samples for comparable groups, 3) manually inspecting data from all participants for quality using independent measures taken from raters who were blind to the hypotheses of the experiment, and 4) setting up detailed instructions and checks throughout the experiment to promote participant engagement (details provided in the Method section). Despite our best efforts, it is likely there are limitations that we have not considered or perfectly controlled for in our experiment. For example, we were unable to collect in-person data from older adults during the project period, largely due to increased health risks associated with bringing older adults to campus for in-person testing during the global pandemic. Thus, our older adult data exclusively came from online sources. In general, online older adult samples tend to perform better on several cognitive measures (e.g., verbal fluency, subjective memory, subjective health, etc.)^59^, which may result in weaker age-effects (specifically less evidence of age-related cognitive decline) and reduced potential for generalizability of our findings. However, in our study, we are still able to see significant age-related effects in the expected patterns (i.e., evidence of age-related deficits in performance) across our variables and analyses of interest, such as age-related increases in perseveration, and age-related decline in sensitivity to reinforcement learning. We expect that these age-related effects would only get stronger in a more diverse sample. Furthermore, by incorporating an independent and more standardized measure of cognitive functioning such as the MST, we are able to capture unique individual differences in cognitive functioning that may also be influencing performance on our decision-making task. With that said, future studies using this task could aim to collect a more representative and heterogeneous older adult population, with age-related cognitive decline characterized using standard neuropsychological and physiological measures, to strengthen the generalizability of our findings.

In sum, the finding that memory and reinforcement learning each exhibit distinct patterns with age and age-related cognitive decline provides new insights into the computational basis of age-related differences in decision-making, and suggests several new avenues for further research that may yield interventions of importance for mitigating the harmful effects of cognitive aging on individuals and their communities.

## Data Availability

All de-identified data and the final versions of all MATLAB model and analysis code, as well as R scripts used to generate each data figure, will be made freely available at the UCI CCNL GitHub repository (https://github.com/uciccnl) upon publication.
